# Adopting a High-Polyphenolic Diet Is Associated with an Improved Glucose Profile: Prospective Analysis within the PREDIMED-Plus Trial

**DOI:** 10.3390/antiox11020316

**Published:** 2022-02-04

**Authors:** Anna Tresserra-Rimbau, Sara Castro-Barquero, Nerea Becerra-Tomás, Nancy Babio, Miguel Ángel Martínez-González, Dolores Corella, Montserrat Fitó, Dora Romaguera, Jesús Vioque, Angel M. Alonso-Gomez, Julia Wärnberg, José Alfredo Martínez, Luís Serra-Majem, Ramon Estruch, Francisco J. Tinahones, José Lapetra, Xavier Pintó, Josep A. Tur, José López-Miranda, Naomi Cano-Ibáñez, Miguel Delgado-Rodríguez, Pilar Matía-Martín, Lidia Daimiel, Vicente Martín Sánchez, Josep Vidal, Clotilde Vázquez, Emili Ros, Francisco Javier Basterra, María Fernández de la Puente, Eva M. Asensio, Olga Castañer, Vanessa Bullón-Vela, Lucas Tojal-Sierra, Enrique Gómez-Gracia, Eugenio Cases-Pérez, Jadwiga Konieczna, Antonio García-Ríos, Tamara Casañas-Quintana, María Rosa Bernal-Lopez, José Manuel Santos-Lozano, Virginia Esteve-Luque, Cristina Bouzas, Zenaida Vázquez-Ruiz, Antoni Palau-Galindo, Rocio Barragan, Mercè López Grau, Cristina Razquín, Leire Goicolea-Güemez, Estefanía Toledo, Manel Vila Vergaz, Rosa M. Lamuela-Raventós, Jordi Salas-Salvadó

**Affiliations:** 1 Department of Nutrition, Food Science and Gastronomy, XIA, School of Pharmacy and Food Sciences, INSA, University of Barcelona, 08921 Barcelona, Spain; lamuela@ub.edu; 2 Centro de Investigación Biomédica en Red Fisiopatología de la Obesidad y la Nutrición (CIBEROBN), Institute of Health Carlos III, 28029 Madrid, Spain; sacastro@clinic.cat (S.C.-B.); n.becerra-tomas@imperial.ac.uk (N.B.-T.); nancy.babio@urv.cat (N.B.); mamartinez@unav.es (M.Á.M.-G.); dolores.corella@uv.es (D.C.); mfito@imim.es (M.F.); mariaadoracion.romaguera@ssib.es (D.R.); angelmaria.alonsogomez@osakidetza.eus (A.M.A.-G.); jwarnberg@uma.es (J.W.); jalfmtz@unav.es (J.A.M.); lserra@dcc.ulpgc.es (L.S.-M.); restruch@clinic.cat (R.E.); fjtinahones@uma.es (F.J.T.); jose.lapetra.sspa@juntadeandalucia.es (J.L.); xpinto@bellvitgehospital.cat (X.P.); pep.tur@uib.es (J.A.T.); jlopezmir@uco.es (J.L.-M.); clotilde.vazquez@fjd.es (C.V.); eros@clinic.ub.es (E.R.); fj.basterra.gortari@navarra.es (F.J.B.); maria.fernandezdelapuente@urv.cat (M.F.d.l.P.); eva.m.asensio@uv.es (E.M.A.); ocastaner@imim.es (O.C.); lucas.tojalsierra@osakidetza.eus (L.T.-S.); egomezgracia@uma.es (E.G.-G.); jadwiga.konieczna@ssib.es (J.K.); h72garia@uco.es (A.G.-R.); t.casanas@adventiapharma.com (T.C.-Q.); rosa.bernal@ibima.eu (M.R.B.-L.); jsantos11@us.es (J.M.S.-L.); cristina.bouzas@uib.es (C.B.); zvazquez@unav.es (Z.V.-R.); rocio.barragan@uv.es (R.B.); mercelopez.bcn.ics@gencat.cat (M.L.G.); crazquin@unav.es (C.R.); LEIRE.GOICOLEAGUEeMEZ@osakidetza.eus (L.G.-G.); etoledo@unav.es (E.T.); mvilav.bcn.ics@gencat.cat (M.V.V.); jordi.salas@urv.cat (J.S.-S.); 3 Department of Internal Medicine, Institut d’Investigacions Biomèdiques August Pi Sunyer (IDIBAPS), Hospital Clinic, University of Barcelona, 08036 Barcelona, Spain; 4 Department of Epidemiology and Biostatistics, School of Public Health, Imperial College London, London W2 1PG, UK; 5 Unitat de Nutrició, Departament de Bioquímica i Biotecnologia, Universitat Rovira i Virgili, 43204 Reus, Spain; antonio.palau@urv.cat; 6 Nutrition Unit, University Hospital of Sant Joan de Reus, 43204 Reus, Spain; 7 Institut d’Investigació Sanitària Pere Virgili (IISPV), 43204 Reus, Spain; 8 Department of Preventive Medicine and Public Health, University of Navarra, IDISNA, 31008 Pamplona, Spain; 9 Department of Nutrition, Harvard T.H. Chan School of Public Health, Boston, MA 02115, USA; 10 Department of Preventive Medicine, University of Valencia, 46010 Valencia, Spain; 11 Unit of Cardiovascular Risk and Nutrition, Institut Hospital del Mar de Investigaciones Médicas Municipal d’Investigació Médica (IMIM), 08007 Barcelona, Spain; 12 Research Group on Nutritional Epidemiology & Cardiovascular Physiopathology (NUTRECOR), Health Research Institute of the Balearic Islands (IdISBa), 07120 Palma de Mallorca, Spain; 13 CIBER de Epidemiología y Salud Pública (CIBERESP), Instituto de Salud Carlos III, 28029 Madrid, Spain; vioque@umh.es (J.V.); ncanojim7@alumnes.ub.edu (N.C.-I.); mdelgado@ujaen.es (M.D.-R.); vicente.martin@unileon.es (V.M.S.); 14 Alicante Institute for Health and Biomedical Research, University Miguel Hernandez (ISABIAL-UMH), 03010 Alicante, Spain; 15 Bioaraba Health Research Institute, Osakidetza Basque Health Service, Araba University Hospital, University of the Basque Country UPV/EHU, 01009 Vitoria-Gasteiz, Spain; 16 Department of Nursing, Institute of Biomedical Research in Málaga (IBIMA), University of Málaga, 29010 Malaga, Spain; 17 Department of Nutrition, Food Sciences, and Physiology, Center for Nutrition Research, University of Navarra, 31008 Pamplona, Spain; mbullon@alumni.unav.es; 18 Cardiometabolic Nutrition Group, IMDEA Food, CEI UAM + CSIC, 28049 Madrid, Spain; 19 Research Institute of Biomedical and Health Sciences (IUIBS), University of Las Palmas de Gran Canaria & Centro Hospitalario Universitario Insular Materno Infantil (CHUIMI), Canarian Health Service, 35016 Las Palmas de Gran Canaria, Spain; 20 Department of Internal Medicine, Regional University Hospital of Malaga, Instituto de Investigación Biomédica de Malaga (IBIMA), University of Malaga, 29010 Malaga, Spain; 21 Department of Family Medicine, Research Unit, Distrito Sanitario Atención Primaria Sevilla, 41010 Sevilla, Spain; 22 Lipids and Vascular Risk Unit, Internal Medicine, Hospital Universitario de Bellvitge, 08908 Hospitalet de Llobregat, Spain; vestevlu9@alumnes.ub.edu; 23 Research Group on Community Nutrition & Oxidative Stress, University of Balearic Islands-IUNICS, 07122 Palma de Mallorca, Spain; 24 Department of Internal Medicine, Maimonides Biomedical Research Institute of Cordoba (IMIBIC), Reina Sofia University Hospital, University of Cordoba, 14004 Cordoba, Spain; 25 Department of Preventive Medicine and Public Health, University of Granada, 18016 Granada, Spain; 26 Instituto de Investigación Biosanitaria, Complejo Hospitales Universitarios de Granada, Universidad de Granada, 18016 Granada, Spain; 27 Division of Preventive Medicine, Faculty of Medicine, University of Jaén, 23071 Jaen, Spain; 28 Department of Endocrinology and Nutrition, Instituto de Investigación Sanitaria Hospital Clínico San Carlos (IdISSC), 28040 Madrid, Spain; mmatia@ucm.es; 29 Nutritional Control of the Epigenome Group, Precision Nutrition and Obesity Program, IMDEA Food, CEI UAM + CSIC, 28029 Madrid, Spain; lidia.daimiel@imdea.org; 30 Institute of Biomedicine (IBIOMED), University of León, 24071 Leon, Spain; 31 CIBER Diabetes y Enfermedades Metabólicas (CIBERDEM), Instituto de Salud Carlos III (ISCIII), 28029 Madrid, Spain; jovidal@clinic.cat; 32 Department of Endocrinology, Institut d’Investigacions Biomédiques August Pi Sunyer (IDIBAPS), Hospital Clinic, University of Barcelona, 08036 Barcelona, Spain; 33 Department of Endocrinology and Nutrition, Hospital Fundación Jimenez Díaz, Instituto de Investigaciones Biomédicas IISFJD, University Autonoma, 28040 Madrid, Spain; 34 Lipid Clinic, Department of Endocrinology and Nutrition, Institut d’Investigacions Biomèdiques August Pi Sunyer (IDIBAPS), Hospital Clínic, 08036 Barcelona, Spain; 35 Department of Preventive Medicine and Public Health, Instituto de Investigación Biomédica de Málaga-IBIMA, School of Medicine, University of Málaga, 29071 Malaga, Spain; 36 Centro de Salud Raval, 03203 Alicante, Spain; ecases@coma.es; 37 ABS Reus V. Centre d’Assistència Primària Marià Fortuny, SAGESSA, 43205 Reus, Spain

**Keywords:** antioxidants, Mediterranean diet, flavonoids, phenolic acids, obesity, glucose, HbA1c, glycosylated hemoglobin, metabolic syndrome

## Abstract

Previous studies suggested that dietary polyphenols could reduce the incidence and complications of type-2 diabetes (T2D); although the evidence is still limited and inconsistent. This work analyzes whether changing to a diet with a higher polyphenolic content is associated with an improved glucose profile. At baseline, and at 1 year of follow-up visits, 5921 participants (mean age 65.0 ± 4.9, 48.2% women) who had overweight/obesity and metabolic syndrome filled out a validated 143-item semi-quantitative food frequency questionnaire (FFQ), from which polyphenol intakes were calculated. Energy-adjusted total polyphenols and subclasses were categorized in tertiles of changes. Linear mixed-effect models with random intercepts (the recruitment centers) were used to assess associations between changes in polyphenol subclasses intake and 1-year plasma glucose or glycosylated hemoglobin (HbA1c) levels. Increments in total polyphenol intake and some classes were inversely associated with better glucose levels and HbA1c after one year of follow-up. These associations were modified when the analyses were run considering diabetes status separately. To our knowledge, this is the first study to assess the relationship between changes in the intake of all polyphenolic groups and T2D-related parameters in a senior population with T2D or at high-risk of developing T2D.

## 1. Introduction

The prevalence of diabetes is experiencing an increasing trend, and in 2019 it was the ninth leading cause of death in the world. Additionally, individuals with diabetes are more likely to suffer from other noncommunicable diseases such as heart attacks, strokes, or kidney disease. The expectations for the forthcoming years are not encouraging since the prevalence of diabetes has been increasing over the past decades. Nevertheless, type-2 diabetes (T2D), the most prevalent type, can be prevented by modifying harmful behavioral risk factors such as smoking, an unhealthy diet, sedentarism, and alcohol abuse [1]. In the search for the best dietary pattern to prevent or stop the progression of T2D, plant-based diets such as Mediterranean-style, vegetarian or vegan diets have been studied in several prospective observational studies and clinical trials [2].

Healthy plant-based diets are based on the consumption of large amounts of whole grains, fruits, vegetables, legumes, and nuts, as well as healthy fats such as extra virgin olive oil, which are associated with a lower risk of developing cardiovascular disease and T2D [3]. A trait all these foods have in common is a richness in polyphenols, bioactive plant secondary metabolites with a vast structural diversity. According to their structure, polyphenols are classified into two main groups: flavonoids and non-flavonoids. Polyphenols in the flavonoid group share the C6-C3-C6 structure and can be divided into the following subgroups: flavones, flavonols, theaflavins, catechins, proanthocyanidins (polymeric forms), flavanones, anthocyanidins, and isoflavones, whereas the non-flavanoids are classified as phenolic acids, lignans, and stilbenes [4].

Protective effects of polyphenols against the incidence and complications of T2D are supported by mechanistic studies conducted in animals [5] as well as clinical and epidemiological studies [6], although the available evidence is still limited and inconsistent. Furthermore, no previous study has examined the association between changes in the intake of all polyphenolic groups and subgroups and T2D-related parameters in a population with or at high-risk of T2D. The aim of the present work was to determine whether changing to a high polyphenol diet is associated with an improved glucose profile. Due to the heterogeneity of polyphenols in terms of bioavailability and metabolism, they were studied in separate groups. 

## 2. Materials and Methods

### 2.1. Study Design and Participants

The present study is a prospective cohort analysis conducted in the context of the PREDIMED-Plus trial [7,8], an ongoing six-year multicenter, parallel group, randomized, lifestyle intervention study involving 6874 participants enrolled in 23 recruitment centers in Spain from October 2013 to December 2016. Eligible participants were men (aged 55–75 years) and women (aged 60–75 years) with a body mass index (BMI) between 27 and 40 kg/m^2^ and the presence of three or more components of metabolic syndrome (updated harmonized criteria of the International Diabetes Federation and the American Heart Association and National Heart, Lung and Blood Institute) [9].

Participants were randomly assigned, in a 1:1 ratio, to one of two groups: an intensive weight-loss intervention group (based on an energy-restricted Mediterranean diet, individualized physical activity plan, and behavioral support) or a control group (based on the traditional Mediterranean diet and usual health care). The detailed study protocol and eligible and exclusion criteria can be found elsewhere [8,10], including at http://predimedplus.com (accessed on 10 January 2022).

**Figure 1:**
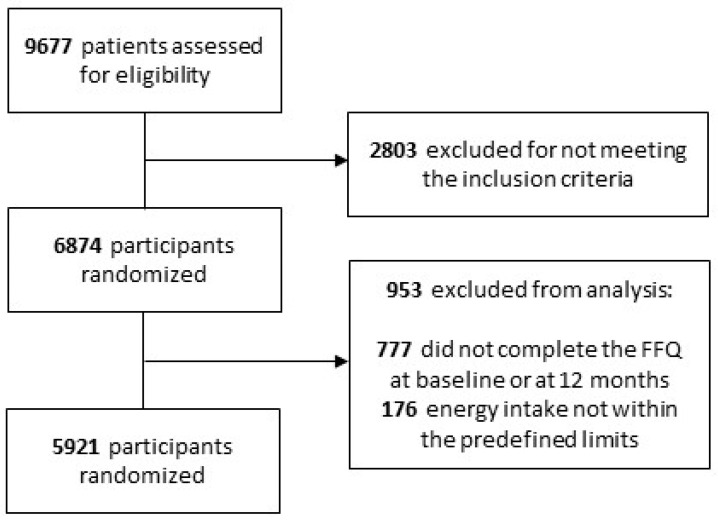
Flow chart of the participants.

For the present analysis, 777 participants with missing dietary data and 176 with extreme energy intakes (<500 or >3500 for women and <800 and >4000 for men) [9] either at baseline or at the annual visit were excluded. Consequently, a total of 5921 participants were available for the analysis (Figure 1).

### 2.2. Dietary Assessment and Polyphenol Intake

At baseline, and at one year of follow-up visits, registered dietitians collected data on dietary intake using a validated 143-item semi-quantitative food-frequency questionnaire (FFQ) [11], from which the total energy and nutrient intake were calculated based on Spanish food composition tables [12]. Additionally, a validated 17-point score questionnaire on adherence to an energy-restricted traditional Mediterranean diet was filled out [13].

The 143-item FFQ was also used to calculate polyphenol intake together with the Phenol-Explorer database (www.phenol-explorer.eu (accessed on 15 September 2021)). Individual polyphenol intakes were obtained by multiplying the content of each polyphenol in each food item with polyphenols (mg/g) by the daily consumption of this food item (g/day) and then summing the product across all food items. Total polyphenols and polyphenol subclasses were then adjusted for total energy intake using the residual method [14], and variables were transformed into tertiles of changes (one year vs. baseline).

### 2.3. Ascertainment of the Endpoints

The main endpoints were one-year changes of fasting plasma glucose (mg/dL) and glycosylated hemoglobin (HbA1c) (%) levels. Both parameters were measured in overnight fasting blood samples by routine laboratory tests.

### 2.4. Assessment of Covariates

Participants filled out a general questionnaire to provide data on lifestyle habits, education, concurrent diseases, and medication use. Physical activity was measured by a Regicor Short Physical Activity Questionnaire validated for the Spanish population [15].

Anthropometric parameters were measured at baseline and every follow-up visit by trained dietitians according to the PREDIMED-Plus protocol. Height, weight, waist, and hip circumference were measured in duplicate by trained staff. BMI was calculated as weight in kilograms divided by height in meters squared.

Blood samples were collected after overnight fasting and stored frozen (−80 °C). Serum triglyceride and total and high-density lipoprotein (HDL) cholesterol levels were measured by routine laboratory tests using standard enzymatic methods.

Sociodemographic and lifestyle variables were categorized in four categories as follows: education (primary, secondary, or high school), physical activity (sedentary, moderately active, and active), smoking status (never, former, or current smoker), and BMI (27.0–29.9, 30.0–34.9, or ≥35 kg/m^2^).

Previous diagnosis of T2D was also registered, as well as glucose-lowering treatment. T2D was diagnosed according to American Diabetes Association guidelines: fasting plasma glucose levels ≥ 7.0 mmol/L (≥126 mg/dL), HbA1c levels ≥ 6.5% or 2 h plasma glucose levels ≥ 11.1 mmol/L (≥200.0 mg/dL) after an oral dose of 75 g glucose [13]. Prediabetes was defined according to the criteria of the American Diabetes Association as impaired fasting glucose (5.6–6.9 mmol/L, or 100–125 mg/dL) and/or raised HbA1c of 39–47 mmol/mol (5.7–6.4%) [16].

### 2.5. Statistical Analyses

Baseline characteristics according to tertiles of changes in total polyphenol intake are presented as means (±SD) for quantitative variables and frequencies for categorical variables. One-factor ANOVA tests were used to assess the differences between tertiles and chi square tests for categorical variables.

Linear mixed-effect models with random intercepts at the recruitment center and cluster family level were used to assess associations between changes in polyphenol subclasses intake and glucose and HbA1c levels over the first year of follow-up. The intake of total polyphenols and the main polyphenol subclasses were distributed into tertiles of changes in consumption after one year of follow-up. To assess the linear trend (*p* for trend) across tertiles of polyphenol intake, the mean value was assigned to each tertile. Model 1 was minimally adjusted for age, sex, and study arm. Model 2 was additionally adjusted for smoking status and levels of education and physical activity at baseline (all categorical). Model 3 was further adjusted for baseline variables such as BMI, energy intake, and intakes of carbohydrates, protein, saturated fatty acids, and alcohol (continuous), and glucose-lowering treatment (Yes/No).

To account for multiple comparisons, we applied the Bonferroni correction to interpret the results. Considering the 12 polyphenols analyzed, significance was established at a *p* value threshold of 0.004 (*p* value < 0.05/12 = 0.004), although all *p* values below 0.05 have been mentioned. Statistical analyses were performed using STATA software (version 16; StataCorp, College Station, TX, USA), and statistical significance was set at *p* < 0.05. We used the PREDIMED-Plus longitudinal database generated on 26 June 2020 (202006290731_PREDIMEDplus).

## 3. Results

This work involved 5921 participants from the PREDIMED-plus cohort that completed the first year of the study. The mean age of the population was 65.0 ± 4.9 years, and 48.2% were women; 30.7% had been diagnosed with diabetes at baseline, and 48.5% were prediabetic. The mean total polyphenol intake was 854 ± 318 mg/day at baseline and 855.0 ± 293 mg/day after one year, indicating no overall change. Breaking down the polyphenols by type, 58% corresponded to flavonoids, 33% were phenolic acids, and the rest were stilbenes, lignans and others, which remained the same after one year. Hydroxycinnamic acids were the most consumed polyphenol class (30%), followed by flavanols (27%), proanthocyanidins (24%), flavanones (10.6%), flavones (9%), flavonols (6%), anthocyanidins (5%), catechins (3%) and hydroxybenzoic acids (2%).

Table 1 summarizes the baseline characteristics of participants classified in tertiles according to changes in total polyphenol intake adjusted for energy using the residual method. During the first year, participants in the lowest tertile (T1) reduced their polyphenol intake by a mean of 249 mg/day, whereas those in the highest tertile (T3) increased their intake by a mean of 256 mg/day. The intake in the middle tertile (T2) remained quite stable. T3 included the highest percentage of men and participants from the intervention group (energy-restricted Mediterranean diet plus physical activity). No significant differences across tertiles were observed regarding age, educational level, smoking habit, physical activity, diabetes status, glucose and HbA1c levels at baseline, and all groups had lower levels of glucose and HbA1c after one year. This is due to the interventions that all participants received, which were (1) an intensive weight-loss intervention based on an energy-restricted Mediterranean diet, individualized physical activity plan, and behavioral support or (2) an intervention based on the traditional Mediterranean diet and usual health care (control group). According to the Mediterranean Diet score, participants from all groups had healthier diets after one year. Although, the greatest reduction in glucose was observed among the participants in T3, that is, those who adopted a high polyphenol diet. It is worth mentioning that fasting glucose and HbA1c levels were also significantly lower in T1. This could be explained because participants were divided in tertiles of change of polyphenol intake, but not all variables across the groups were equally distributed. For instance, the ones who decreased polyphenol intake after one year also had the highest consumption of olive oil. Therefore, the real associations appeared after the statistical models were adjusted for confounders. 

Table 2 shows dietary changes after one year corresponding to each tertile of changes in total polyphenol intake. Although all the participants adopted healthier dietary patterns, there were differences between groups. For instance, those in T3 reduced their total calory intake per day by almost 200 kcal, compared to 129 kcal in T1. This difference can be explained by the higher reduction in dietary protein and saturated fatty acids in T3. Nevertheless, the most notable reduction in alcohol intake was in T1. We observed that participants who reduced their total polyphenol consumption also had lower carbohydrate and higher MUFA and PUFA intakes. The improvements in these parameters seem to be correlated with changes in diet, as these participants reduced their consumption of cookies, pastries, and fruit. Overall, participants in T3 obtained the highest score in the Mediterranean-diet adherence test after one year, and they had consumed more vegetables, fruits, and fiber and fewer cereals, dairy, meat, and sugary items (cookies, pastries and sweets, sugar, and soft drinks). No differences were observed for fish and nuts.

We generated linear mixed models to study the association between changes in glucose and HbA1c levels and tertiles of change in polyphenol intake after one year (Table 3). Analyses were performed for total polyphenols, total flavonoids (including anthocyanidins, catechins, proanthocyanidins, flavanones, flavones, and flavonols), total phenolic acids (including hydroxycinnamic acids and hydroxybenzoic acids), lignans, and stilbenes. We compared the participants in T1 and T3 using T2 as a reference, as the polyphenol intake in this group did not change. The extreme groups were also compared with each other (T1 vs. T3).

In multivariable-adjusted models, considering anthropometric, sociodemographic, lifestyle, and dietary variables after one year of follow-up, an increment in total polyphenol intake was inversely associated with glucose levels (β = −1.76; 95% CI −3.18, −0.34) when comparing T3 with T2. Moreover, HbA1c values were lower in T1 than in T2 (β = −0.039; 95% CI −0.076, −0.002), although further analyses revealed that this result was correlated with the hydroxycinnamic acid intake (β = −0.04; 95% CI −0.077, −0.004; T1 vs. T2).

Due to the heterogeneity of polyphenols, they were studied separately. The increase in total flavonoids was also correlated with a decrease in glucose levels (β = −1.56; 95% CI −2.99, −0.13; T3 vs. T1). Among the flavonoids, flavones and flavonols were both inversely associated with glucose and HbA1c, the last with a lineal relationship. Anthocyanidins were also inversely associated with HbA1c (β = −0.037; 95% CI −0.075, 0.000; T3 vs. T2), but, after adjusting for all the potential confounding variables, the association was not significant (*p* = 0.05). Some correlations with glucose and HbA1c were also found for the non-flavonoids: lignans and stilbenes. In the case of lignans, the association was also linear.

**Figure 2:**
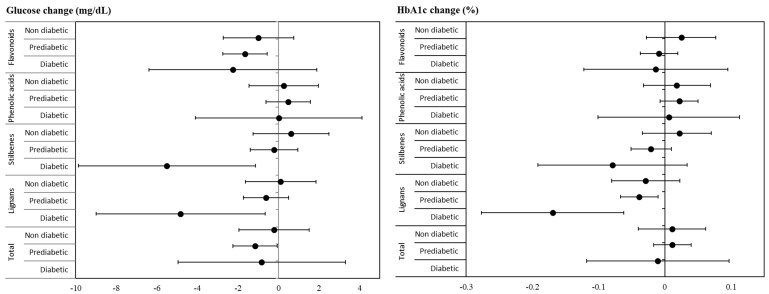
Glucose (mg/dL) and glycosylated hemoglobin (%) changes after one year (β; 95% CI) comparing extreme tertiles of energy-adjusted polyphenol intake by diabetes status at baseline. Results from fully adjusted linear mixed models.

We wanted to study if diabetes status was an important factor when analyzing the impact of polyphenol intake on glucose and HbA1c, so the analyses were repeated after dividing the population in three groups: those without diabetes, and prediabetic and diabetic participants (Table 4 and Figure 2). Interestingly, the role of polyphenols was found to differ considerably depending on the diabetes status. No significant associations were found within the non-diabetic group, whereas the participants who most benefited from a higher polyphenol intake were prediabetic. In this group, several polyphenol classes were inversely associated with levels of glucose (total polyphenols, total flavonoids, proanthocyanidins, flavanones, and flavones) or HbA1c (flavones and lignans). Once again, hydroxycinnamic acid intake was directly associated with HbA1c. Fewer polyphenol groups were associated with glucose-related parameters in diabetic participants (flavonols, lignans, and stilbenes).

## 4. Discussion

This work shows a longitudinal inverse association between certain classes of polyphenols and levels of glucose and HbA1c in the PREDIMED-Plus cohort after one year of follow-up. To our knowledge, this is the first study to assess the relationship between changes in the intake of all polyphenol groups and T2D-related parameters in a senior population with or at high risk of T2D.

Although evidence is still limited, it has been suggested that the benefits of dietary polyphenols regarding T2D may include anti-inflammatory, antioxidant, and glucose metabolism regulatory effects, such as the inhibition of α-amylases and α-glucosidases, protection against glucose toxicity in pancreatic β-cells [17], and modulation of glucose transporter type-4 (GLUT4) receptors.

### 4.1. Anthocyanidins

Anthocyanidins are a subtype of flavonoids responsible for many of the red to violet colors in fruits and vegetables. The main food sources of anthocyanins are berries, including grapes and derivatives such as wine [4]. We found a significant inverse association between changes in anthocyanin intake and HbA1c levels (T3 vs. T2), although it was insignificant for glucose levels. In line with this result, a 12-week randomized double blind placebo-controlled trial showed that daily supplementation of 320 mg of anthocyanidins in 160 prediabetic participants significantly reduced HbA1c, while no significant changes were observed in glucose levels [18]. In the same study population, a higher anthocyanin intake was correlated with a lower prevalence of T2D in overweight men [19]. Moreover, in a meta-analysis of cohort studies, Guo et al. showed a 5% decrease in T2D risk with each 7.5 mg/day increment in anthocyanin intake [20].

### 4.2. Proanthocyanidins

Proanthocyanidins are classified as flavanols, together with catechins and theaflavins [4]. An increase in proanthocyanidin intake was associated with a decrease in fasting glucose and HbA1c levels, although not in the fully adjusted model. In the stratified analyses no significant results were observed, except for fasting glucose levels in prediabetic participants. This was in line with the null effects observed in clinical trials administering proanthocyanidin supplements in T2D patients [21,22], whereas some evidence suggests this flavanol can improve insulin resistance [23]. Although previous studies with the same population found inverse associations between proanthocyanidin and catechin intake and T2D risk, in the present work only proanthocyanidins had an effect on T2D indicators [19].

### 4.3. Flavones

Flavones were inversely associated with fasting glucose levels and HbA1c in prediabetic participants. The main food sources of flavones in the study population were whole grain products, bread, and oranges. No associations between flavones and T2D risk were previously found in the same study population or a similar cohort at high risk of cardiovascular disease [19,24].

### 4.4. Flavonols

In the case of flavonols, the main dietary sources were red wine and vegetables such as onion, spinach, and lettuce. After one year of follow-up, a significant increase in vegetable consumption was observed, especially in participants with a higher intake of dietary polyphenols. Changes in flavonol intake were inversely associated with changes in HbA1c levels in both prediabetic and diabetic participants, which is in line with the antidiabetic effects of flavonols postulated by two large observational studies [25,26]. However, in the present study, an increase in flavonol intake was significantly correlated with higher fasting glucose levels, although the correlation was not significant in the stratified analysis. These findings agree with previous observations for T2D risk in the same cohort [19].

### 4.5. Hydroxybenzoic and Hydroxycinnamic Acids

Total phenolic acid intake was not associated with T2D parameters, yet interestingly, the intake of hydroxycinnamic acids was directly associated with an increase in fasting glucose and HbA1c levels, except in the fully adjusted model, and the highest increase in HbA1c levels was observed in prediabetic participants. Hydroxycinnamic acids accounted for more than 90% of the phenolic acid intake, coffee being the main food source. In contrast, in a prospective analysis of 4923 T2D participants, drinking two or more cups of coffee per day was associated with a 41% reduction in all-cause mortality risk [27]. Similar findings were reported in a meta-analysis of ten prospective cohort studies, where the risk for all-cause mortality was reduced in a coffee-consuming T2D population [28]. It should be stressed that the antidiabetic properties of coffee are likely to be mediated by the polyphenol content rather than caffeine [29,30]. No significant association was observed for hydroxybenzoic acids; among them, ellagic acid has been correlated with lower HbA1c and fasting glucose levels and is reported to promote insulin secretion [31].

### 4.6. Lignans

Even though the ingestion of lignans is low compared to other polyphenol subclasses, its intake has been associated with several health benefits. The main food sources of lignans in this cohort were fiber-rich foods, such as whole grain cereals and olive oil. In the present analysis, a higher intake of fiber was observed in participants who increased their dietary polyphenol intake after one year of follow-up. Those with the highest increase in lignan intake, especially prediabetic and diabetic participants, had lower levels of fasting glucose and HbA1c in the fully adjusted model. These results agree with previous findings in the PREDIMED cohort and in two U.S. women cohorts [32]. Dietary lignan intake has been linked with improved glycemic control, mainly HbA1c and fasting plasma glucose levels, but the evidence from observational studies assessing its effect on T2D risk is limited [33,34,35]. The antidiabetic effects exerted by lignans may be mediated by improvements in central obesity [36]. Other potential mechanisms of action include an inhibition of α-amylase and α-glucosidase, improvements in insulin sensitivity, activation of AMPk and GLUT4 receptors, and acting as antagonists of adiponectin receptors [37]. Associated improvements in fasting plasma glucose levels in non-diabetic patients have also been observed [34].

### 4.7. Stilbenes

Stilbenes, mainly resveratrol, have been previously associated with a lower risk of T2D in the PREDIMED cohort [24]. However, in the present study changes in stilbene intake showed only a mild inverse association with alterations in fasting plasma and HbA1c levels, which was stronger in diabetic participants. Lui et al. performed a meta-analysis of 11 controlled trials administering trans resveratrol in overweight or obese individuals to assess whether its consumption affected glycemic status or insulin sensitivity [38]. Notably, in alignment with our findings, non-significant effects on glycemic measurements were observed in non-diabetic participants. The main food source of stilbenes is red wine, and its moderate intake has been associated with a lower risk of T2D [39].

### 4.8. Effect Modification by Diabetes Status

The improvements in HbA1c levels observed in the present study are similar to those arising from other dietary interventions in T2D patients, such as high-fiber diets or health education programs [10,40]. According to the United States Food and Drug Administration, even a modest reduction in HbA1c levels (0.3 to 0.4%) reduces the risk of developing diabetes [41].

Polyphenol intake has been shown to have a modulatory effect on the gut microbiota profile [42]. It is also recognized that the gut microbiota plays a key role in the development of T2D, due to its implication in carbohydrate metabolism. Moreover, intestinal dysbiosis has been described in both T2D and prediabetic patients, indicating that certain compositional changes in the microbiota participate in the development of the disease [43,44]. Another potential mechanism underlying the antidiabetic effect of polyphenols is the induction of GLP-1 secretion [45]. The GLP-1 signaling pathway has been extensively explored to develop effective therapies for T2D, and emerging evidence shows that some phenolic compounds can stimulate GLP-1 secretion from intestinal L-cells and may, therefore, be helpful in improving glucose homeostasis [45].

### 4.9. Strengths and Limitations

The limitations of this study are mostly related to the estimation of polyphenol intake and the confounding variables, as residual confounding factors may still be present. Regarding polyphenols, although we used the most updated and comprehensive database available (Phenol-explorer), not all foods from the FFQ were included in the database (e.g., honey), and the questionnaire does not cover all polyphenol-rich foods (e.g., spices) or the polymeric, non-extractable polyphenols associated with cell wall macromolecules. Furthermore, phenolic intake can be affected by the variable polyphenol content in foods, which depends on ripeness, environmental factors, processing and storage, and variety [4]. Finally, bioavailability was not considered, and the results might not be generalizable to different populations.

The main strengths of the study are the large sample size, the multicenter design, and longitudinal approach. Regarding sociodemographic and lifestyle variables (confounders), a standardized protocol was used to reduce the information bias.

## 5. Conclusions

Evidence suggests that a regular consumption of dietary polyphenols is associated with improvements in essential biological outcomes for T2D prevention and management. However, assessing the health benefits of polyphenol intake is complex due to their diverse chemical structure and variable bioavailability, the complexity of estimating their content in foods and therefore their intake, potential interactions with other nutrients or polyphenols, and biological aspects that may modify metabolization [46]. Even though the protective role of dietary polyphenols in health has been widely demonstrated, more randomized clinical trials are needed to clarify how their consumption affects biomarkers related to T2D. Moreover, more information is needed to determine which polyphenol subclasses are the most beneficial and which food sources produce the best results in terms of T2D prevention and management.

## Figures and Tables

**Table 1:** Characteristics of the study participants, according to tertiles of changes in total polyphenol intake.

	Tertiles of Δ Polyphenol Intake after 1 Year			
Change of polyphenol intake (mg/day), median (min to max)	T1 −249 (−2400 to −106)	T2 1.52 (−106 to 107)	T3 256 (107 to 1400)	P
No. of subjects	1974	1974	1973	
Allocated in the intervention group	901 (45.6)	955 (48.4)	1044 (52.9)	<0.001
Age (years), mean ± SD	65.2 ± 4.9	65.0 ± 4.8	64.9 ± 4.9	0.09
Women, n (%)	987 (50.0)	971 (49.2)	898 (45.5)	0.01
**Education, n (%)**				
Primary school	981 (49.7)	971 (49.2)	990 (50.2)	0.11
High school	545 (27.6)	606 (30.7)	550 (27.9)	
University	448 (22.7)	397 (20.1)	433 (21.9)	
**Current smoker, n (%)**				
Baseline	243 (12.3)	242 (12.2)	250 (12.7)	0.06
After 1 year	205 (11.9)	202 (11.9)	214 (13.3)	0.45
**Physical activity (METS.min/week), mean ± SD**				
Baseline	2543 ± 2283	2454 ± 2329	2527 ± 2353	0.34
1-year change	518 ± 2480 *	506 ± 2363 *	567 ± 2433 *	0.70
**Diabetes status**				
Nondiabetic participants	384 (19.5)	432 (21.9)	415 (21.0)	0.36
Pre-diabetic participants	981 (49.7)	950 (48.1)	941 (47.7)	
Diabetic participants	609 (30.9)	592 (30.0)	617 (31.3)	
**Glucose (mg/dL), mean ± SD**				
Baseline	113.67 ± 28.70	112.18 ± 29.08	114.29 ± 28.97	0.06
1-year change	−2.41 ± 20.92 *	−1.59 ± 23.13	−3.4 ± 23.04 *	0.04
**HbA1c (%), mean ± SD**				
Baseline	6.12 ± 0.87	6.08 ± 0.87	6.11 ± 0.83	0.25
1-year change	−0.09 ± 0.53 *	−0.05 ± 0.53	−0.09 ± 0.57 *	0.07

Values are frequencies and percentages for categorical variables or means ± SDs for continuous variables, except for polyphenol intake, which are median (min-max). Analysis of variance one factor (ANOVA) was used for continuous variables and the χ^2^ test for categorical variables. BMI, body mass index (calculated as weight in kilograms divided by height in meters squared); SD, standard deviation. * Significant differences between baseline and one-year data.

**Table 2:** Changes in daily intake of nutrients, food items, and Mediterranean diet score after one year, according to tertiles of changes in total polyphenol intake.

	Tertiles of Δ Polyphenol Intake after 1 Year				
	T1 −249 (−2400 to −106)	T2 1.52 (−106 to 107)	T3 256 (107 to 1400)	P	Adjusted P
No. of subjects	1974	1974	1973		
Total energy (Kcal/d)					
Baseline	2382 ± 547	2289 ± 541	2430 ± 552	<0.001	<0.001
1-year change	−128.7 ± 524.8	−135.1 ± 483.5	−194.4 ± 541.7	0.002	<0.001
Carbohydrates (g/d)					
Baseline	246 ± 73	231 ± 71	245 ± 73	<0.001	<0.001
1-year change	−34.7 ± 70.0	−29.2 ± 66.0	−31.6 ± 75.6	0.05	<0.001
Fiber (g/d)					
Baseline	29 ± 10	25 ± 8	25 ± 8	<0.001	<0.001
1-year change	0.5 ± 9.7	3.6 ± 8.3	6.8 ± 9.1	<0.001	<0.001
Proteins (g/d)					
Baseline	98 ± 22	96 ± 22	99 ± 22	<0.001	<0.001
1-year change	−1.2 ± 21.8	−2.1 ± 20.8	−4.0 ± 22.1	<0.001	<0.001
MUFA (g/d)					
Baseline	53 ± 16	53 ± 16	56 ± 16	<0.001	<0.001
1-year change	7.0 ± 18.4	4.6 ± 16.8	1.9 ± 18.2	<0.001	<0.001
PUFA (g/d)					
Baseline	18 ± 6	17 ± 6	19 ± 7	<0.001	<0.001
1-year change	1.7 ± 7.2	1.0 ± 6.8	0.1 ± 7.3	<0.001	<0.001
SFA (Kcal/d)					
Baseline	26 ± 9	25 ± 8	27 ± 9	<0.001	<0.001
1-year change	−2.5 ± 8.1	−3.3 ± 7.3	−4.8 ± 8.2	<0.001	<0.001
Alcohol (g/d)					
Baseline	11 ± 14	11 ± 15	12 ± 16	0.05	0.31
1-year change	−2.0 ± 11.3	−1.2 ± 10	−0.5 ± 11.8	<0.001	<0.001
17-points MedDiet score					
Baseline	8.86 ± 2.7	8.45 ± 2.63	8.22 ± 2.65	<0.001	<0.001
1-year change	2.6 ± 3.1	3.3 ± 3.2	3.9 ± 3.3	<0.001	<0.001
**Food items, g/day**					
Vegetables					
Baseline	348 ± 146	326 ± 136	313 ± 127	<0.001	<0.001
1-year change	6.4 ± 153.5	32.0 ± 143.7	60.7 ± 146.8	<0.001	<0.001
Fruits					
Baseline	430 ± 240	341 ± 179	308 ± 167	<0.001	<0.001
1-year change	−58.5 ± 236.0	42.2 ± 177.9	146.9 ± 204.4	<0.001	<0.001
Legumes					
Baseline	21 ± 11	20 ± 11	20 ± 11	0.001	<0.001
1-year change	3.8 ± 13.6	4.6 ± 13.1	4.1 ± 13.3	0.15	0.04
Cereals					
Baseline	146 ± 77	145 ± 74	161 ± 82	<0.001	<0.001
1-year change	−15.4 ± 82.0	−23.1 ± 76.6	−35.2 ± 88.1	<0.001	<0.001
Dairy					
Baseline	348 ± 206	336 ± 193	349 ± 203	0.07	0.05
1-year change	−11.8 ± 191.7	−19.9 ± 177.2	−27.7 ± 193.4	0.03	<0.001
Meat					
Baseline	144 ± 57	146 ± 57	153 ± 61	<0.001	<0.001
1-year change	−8.6 ± 57.1	−16.1 ± 55.3	−24.1 ± 57.0	<0.001	<0.001
Olive oil					
Baseline	39 ± 16	40 ± 17	42 ± 17	<0.001	<0.001
1-year change	6.8 ± 18.9	4.7 ± 17.7	1.4 ± 19.1	<0.001	<0.001
Fish					
Baseline	105 ± 47	101 ± 47	101 ± 47	0.02	0.02
1-year change	8.1 ± 53.0	10.1 ± 50.7	9.7 ± 50.3	0.43	0.30
Nuts					
Baseline	16 ± 17	14 ± 17	15 ± 17	0.04	0.04
1-year change	13.8 ± 23.1	13.3 ± 21.0	14.3 ± 22.1	0.37	0.29
Cookies, pastries, and sweets					
Baseline	30 ± 31	23 ± 27	27 ± 31	<0.001	<0.001
1-year change	−14.7 ± 29.7	−10.4 ± 26.4	−11.6 ± 31.2	<0.001	<0.001
Sugar					
Baseline	6 ± 12	7 ± 12	7 ± 12	0.32	0.62
1-year change	−2.5 ± 9.8	−3.1 ± 10.0	−3.6 ± 10.8	0.003	<0.001
Soft drinks					
Baseline	20 ± 59	21 ± 64	23 ± 66	0.16	0.27
1-year change	−7.0 ± 67.8	−10.4 ± 64.1	−12.9 ± 77.9	0.03	0.004

Values are means ± SD. *p*-values were calculated by ANCOVA tests adjusted for sex, age, intervention group, education level, and recruitment center. MUFA, Monounsaturated Fatty Acids; PUFA, Polyunsaturated Fatty Acids; SFA, Saturated Fatty Acids; MedDiet, Mediterranean Diet.

**Table 3:** Changes in glucose (mg/dL) and glycosylated hemoglobin (HbA1c) (%) according to tertiles of change of polyphenol intake (mg/dL) after one year. Results from linear mixed models.

		T1 (vs. T2)	P	T2	T3 (vs. T2)	P	T3 (vs. T1)	P	P-Trend
	Total polyphenols	−249 (−2400, −106) ^a^		2 (−106, 107)	257 (107, 1400)				
Glucose	Model 1	−2.56 (−3.46, −1.66) ^b^	<0.001	ref.	−2.34 (−3.24, −1.44)	<0.001	0.22 (−0.74, 1.18)	0.66	0.12
	Model 2	−2.78 (−3.72, −1.84)	<0.001	ref.	−2.56 (−3.51, −1.61)	<0.001	0.23 (−0.78, 1.23)	0.62	0.33
	Model 3	−0.94 (−2.36, 0.48)	0.19	ref.	−1.76 (−3.18, −0.34)	0.015	−0.82 (−2.24, 0.61)	0.23	0.43
HbA1c	Model 1	0.015 (−0.008, 0.037)	0.19	ref.	0.008 (−0.015, 0.031)	0.50	−0.007 (−0.031, 0.018)	0.55	0.76
	Model 2	0.011 (−0.012, 0.034)	0.35	ref.	0.001 (−0.023, 0.025)	0.92	−0.01 (−0.035, 0.016)	0.44	0.80
	Model 3	−0.039 (−0.076, −0.002)	0.04	ref.	−0.032 (−0.069, 0.005)	0.09	0.007 (−0.031, 0.044)	0.36	0.46
	Total flavonoids	−195 (−2405, −78)		3 (−78, 84)	193 (84, 1383)				
Glucose	Model 1	0.46 (−0.45, 1.37)	0.32	ref.	−0.6 (−1.51, 0.31)	0.20	−1.06 (−2.04, −0.07)	0.04	0.16
	Model 2	0.01 (−0.95, 0.96)	0.99	ref.	−0.79 (−1.75, 0.18)	0.11	−0.79 (−1.83, 0.24)	0.14	0.16
	Model 3	0.17 (−1.25, 1.59)	0.81	ref.	−1.39 (−2.82, 0.04)	0.06	−1.56 (−2.99, −0.13)	0.03	0.16
HbA1c	Model 1	−0.029 (−0.052, −0–006)	0.014	ref.	−0.033 (−0.057, −0.01)	0.006	−0.004 (−0.029, 0.021)	0.79	0.42
	Model 2	−0.019 (−0.043, 0.005)	0.12	ref.	−0.021 (−0.045, 0.004)	0.09	−0.002 (−0.027, 0.024)	0.95	0.23
	Model 3	−0.024 (−0.061, 0.013)	0.21	ref.	−0.024 (−0.061, 0.013)	0.20	−0.001 (−0.038, 0.037)	0.99	0.72
	Anthocyanidins	−25 (−526, −10)		0 (−10, 10)	25 (10, 209)				
Glucose	Model 1	1.87 (0.96, 2.78)	<0.001	ref.	1.72 (0.81, 2.63)	<0.001	−0.15 (−1.1, 0.8)	0.76	0.05
	Model 2	1.42 (0.46, 2.37)	0.004	ref.	1.74 (0.77, 2.70)	<0.001	0.32 (−0.68, 1.32)	0.56	0.03
	Model 3	0.06 (−1.36, 1.48)	0.93	ref.	−0.43 (−1.86, 1.00)	0.56	−0.49 (−1.92, 0.94)	0.50	0.89
HbA1c	Model 1	0.024 (0.001, 0.047)	0.04	ref.	−0.026 (−0.05, −0.003)	0.03	−0.05 (−0.075, −0.025)	<0.001	0.83
	Model 2	0.019 (−0.004, 0.043)	0.11	ref.	−0.019 (−0.044, 0.005)	0.12	−0.039 (−0.064, −0.013)	0.003	0.35
	Model 3	−0.028 (−0.065, 0.009)	0.14	ref.	−0.037 (−0.075, 0.000)	0.05	−0.009 (−0.047, 0.028)	0.66	0.62
	Catechins	−14 (−162, −5)		0 (−5, 6)	14 (6, 176)				
Glucose	Model 1	−0.46 (−1.35, 0.42)	0.31	ref.	0.25 (−0.65, 1.14)	0.59	0.71 (−0.2, 1.61)	0.12	0.77
	Model 2	0.00 (−0.93, 0.93)	0.99	ref.	0.29 (−0.64, 1.23)	0.54	0.29 (−0.66, 1.24)	0.55	0.51
	Model 3	0.76 (−0.66, 2.17)	0.30	ref.	−0.11 (−1.53, 1.31)	0.90	−0.87 (−2.29, 0.55)	0.23	0.27
HbA1c	Model 1	0.038 (0.016, 0.06)	0.001	ref.	0.029 (0.006, 0.052)	0.01	−0.009 (−0.032, 0.014)	0.36	0.89
	Model 2	0.044 (0.021, 0.067)	<0.001	ref.	0.028 (0.004, 0.051)	0.02	−0.017 (−0.04, 0.007)	0.15	0.90
	Model 3	0.008 (−0.028, 0.045)	0.65	ref.	−0.019 (−0.057, 0.018)	0.30	−0.028 (−0.065, 0.009)	0.14	0.35
	Proanthocyanidins	−122 (−2169, −48)		−4 (−48, 40)	106 (40, 1207)				
Glucose	Model 1	1.97 (1.07, 2.87)	<0.001	ref.	0.27 (−0.63, 1.18)	0.55	−1.7 (−2.64, −0.75)	<0.001	0.10
	Model 2	1.15 (0.19, 2.11)	0.02	ref.	−0.35 (−1.31, 0.61)	0.47	−1.51 (−2.5, −0.51)	0.003	0.01
	Model 3	1.13 (−0.29, 2.55)	0.12	ref.	−0.15 (−1.58, 1.29)	0.84	−1.28 (−2.71, 0.15)	0.08	0.61
HbA1c	Model 1	0.031 (0.008, 0.054)	0.009	ref.	−0.015 (−0.039, 0.008)	0.20	−0.046 (−0.071, −0.022)	<0.001	0.001
	Model 2	0.031 (0.007, 0.055)	0.01	ref.	−0.014 (−0.038, 0.011)	0.28	−0.044 (−0.07, −0.019)	0.001	<0.001
	Model 3	−0.012 (−0.049, 0.025)	0.51	ref.	−0.02 (−0.058, 0.018)	0.30	−0.008 (−0.045, 0.030)	0.70	0.56
	Flavanones	−42 (−554, −5)		13 (−5, 36)	77 (36, 860)				
Glucose	Model 1	−1.48 (−2.38, −0.58)	0.001	ref.	−2.27 (−3.19, −1.35)	<0.001	−0.79 (−1.76, 0.18)	0.11	0.75
	Model 2	−1.37 (−2.31, −0.43)	0.004	ref.	−2.52 (−3.49, −1.55)	<0.001	−1.15 (−2.16, −0.15)	0.04	0.30
	Model 3	0.53 (−0.88, 1.95)	0.46	ref.	−0.33 (−1.75, 1.09)	0.65	−0.86 (−2.28, 0.56)	0.24	0.38
HbA1c	Model 1	−0.026 (−0.048, −0.003)	0.02	ref.	0.019 (−0.004, 0.043)	0.11	0.045 (0.021, 0.07)	<0.001	<0.001
	Model 2	−0.013 (−0.036, 0.010)	0.28	ref.	0.011 (−0.013, 0.036)	0.36	0.024 (−0.001, 0.05)	0.06	0.02
	Model 3	−0.015 (−0.052, 0.022)	0.43	ref.	0.007 (−0.030, 0.044)	0.71	0.022 (−0.015, 0.059)	0.25	0.10
	Flavones	−21 (−305, 1)		13 (1, 30)	55 (30, 344)				
Glucose	Model 1	0.71 (−0.2, 1.63)	0.12	ref.	−0.46 (−1.44, 0.52)	0.36	−1.17 (−2.19, −0.16)	0.02	0.007
	Model 2	1.05 (0.08, 2.02)	0.03	ref.	−0.09 (−1.12, 0.94)	0.86	−1.14 (−2.2, −0.08)	0.03	0.008
	Model 3	−1.2 (−2.64, 0.23)	0.10	ref.	−1.56 (−3.02, −0.11)	0.62	−1.56 (−3.02, −0.11)	0.04	0.20
HbA1c	Model 1	−0.053 (−0.076, −0.029)	<0.001	ref.	−0.026 (−0.051, 0.000)	0.05	0.027 (0.001, 0.053)	0.019	<0.001
	Model 2	−0.045 (−0.07, −0.021)	<0.001	ref.	−0.024 (−0.05, 0.003)	0.08	0.022 (−0.005, 0.049)	0.06	<0.001
	Model 3	−0.015 (−0.053, 0.022)	0.41	ref.	−0.049 (−0.087, −0.012)	0.01	−0.034 (−0.072, 0.004)	0.08	0.12
	Flavonols	−15 (−103, −4)		4 (−4, 12)	26 (12, 177)				
Glucose	Model 1	−1.59 (−2.5, −0.67)	0.001	ref.	1.4 (0.44, 2.36)	0.004	2.99 (1.96, 4.01)	<0.001	0.51
	Model 2	−1.4 (−2.37, −0.44)	0.004	ref.	1.3 (0.3, 2.3)	0.01	2.7 (1.63, 3.78)	<0.001	0.85
	Model 3	0.13 (−1.29, 1.55)	0.86	ref.	−1.36 (−2.78, 0.06)	0.06	−1.49 (−2.93, −0.05)	0.04	0.03
HbA1c	Model 1	0.019 (−0.004, 0.042)	0.11	ref.	−0.053 (−0.077, −0.029)	<0.001	−0.072 (−0.098, −0.045)	<0.001	<0.001
	Model 2	0.017 (−0.007, 0.04)	0.17	ref.	−0.036 (−0.061, −0.011)	0.004	−0.053 (−0.08, −0.026)	<0.001	0.05
	Model 3	−0.005 (−0.042, 0.033)	0.81	ref.	−0.073 (−0.11, −0.036)	<0.001	−0.069 (−0.106, −0.031)	<0.001	0.003
	Hydroxycinnamic acids	−103 (−725, −32)		−3 (−32, 26)	93 (26, 739)				
Glucose	Model 1	−3.07 (−3.96, −2.19)	<0.001	ref.	−1.28 (−2.17, −0.38)	0.005	1.8 (0.86, 2.73)	<0.001	<0.001
	Model 2	−3.24 (−4.17, −2.3)	<0.001	ref.	−1.28 (−2.22, −0.34)	0.007	1.96 (0.97, 2.94)	<0.001	<0.001
	Model 3	−0.94 (−2.36, 0.47)	0.19	ref.	−0.26 (−1.68, 1.16)	0.72	0.69 (−0.74, 2.11)	0.34	0.47
HbA1c	Model 1	−0.015 (−0.037, 0.007)	0.18	ref.	0.02 (−0.003, 0.042)	0.09	0.035 (0.011, 0.059)	0.004	0.41
	Model 2	−0.018 (−0.041, 0.006)	0.14	ref.	0.016 (−0.007, 0.039)	0.18	0.034 (0.009, 0.058)	0.008	0.85
	Model 3	−0.04 (−0.077, −0.004)	0.03	ref.	−0.021 (−0.058, 0.016)	0.26	0.019 (−0.018, 0.057)	0.31	0.40
	Hydroxybenzoic acids	−13 (−55, −7)		−4.4 (−7, −1)	3.3 (−1, 64)				
Glucose	Model 1	0.13 (−0.73, 0.99)	0.77	ref.	−0.06 (−0.94, 0.82)	0.89	−0.19 (−1.14, 0.76)	0.70	0.79
	Model 2	0.27 (−0.63, 1.18)	0.55	ref.	0.04 (−0.88, 0.96)	0.93	−0.23 (−1.22, 0.76)	0.64	0.93
	Model 3	−0.1 (−1.53, 1.33)	0.89	ref.	0.33 (−1.09, 1.76)	0.65	0.44 (−1.02, 1.89)	0.56	0.80
HbA1c	Model 1	−0.038 (−0.06, −0.016)	0.001	ref.	−0.027 (−0.049, −0.004)	0.02	0.011 (−0.013, 0.036)	0.32	0.77
	Model 2	−0.035 (−0.058, −0.013)	0.002	ref.	−0.028 (−0.051, −0.005)	0.016	0.007 (−0.018, 0.032)	0.53	0.70
	Model 3	0.001 (−0.036, 0.038)	0.96	ref.	0.007 (−0.03, 0.044)	0.70	0.006 (−0.032, 0.044)	0.75	0.77
	Lignans	−0.4 (−7,2, −0.1)		0.1 (−0.1, 0.3)	0.5 (0.3, 5.8)				
Glucose	Model 1	0.27 (−0.67, 1.2)	0.58	ref.	0.63 (−0.31, 1.58)	0.19	0.37 (−0.66, 1.4)	0.50	0.05
	Model 2	0.33 (−0.65, 1.3)	0.52	ref.	0.49 (−0.52, 1.49)	0.34	0.16 (−0.91, 1.23)	0.59	0.006
	Model 3	0.54 (−0.89, 1.96)	0.46	ref.	−1.08 (−2.51, 0.35)	0.14	−1.62 (−3.07, −0.17)	0.03	0.08
HbA1c	Model 1	0.025 (0.002, 0.049)	0.03	ref.	0.001 (−0.024, 0.026)	0.96	−0.025 (−0.052, 0.002)	0.06	0.09
	Model 2	0.03 (0.006, 0.054)	0.01	ref.	0.003 (−0.023, 0.029)	0.82	−0.027 (−0.055, 0)	0.04	0.21
	Model 3	0.031 (−0.006, 0.068)	0.10	ref.	−0.041 (−0.078, −0.003)	0.03	−0.072 (−0.11, −0.034)	<0.001	0.003
	Stilbenes	−1.4 (−30.3, −0.6)		0.0 (−0.6, 0.6)	1.7 (0.6, 27.1)				
Glucose	Model 1	−0.54 (−1.43, 0.35)	0.24	ref.	−1.17 (−2.04, −0.3)	0.08	−0.64 (−1.54, 0.27)	0.15	0.004
	Model 2	−0.28 (−1.21, 0.66)	0.46	ref.	−1.09 (−2, −0.18)	0.02	−0.81 (−1.76, 0.14)	0.08	0.008
	Model 3	1.1 (−0.35, 2.55)	0.14	ref.	−0.6 (−2.08, 0.87)	0.42	−1.7 (−3.25, −0.16)	0.03	0.81
HbA1c	Model 1	−0.057 (−0.08, −0.035)	<0.001	ref.	−0.024 (−0.046, −0.002)	0.03	0.033 (0.011, 0.056)	0.004	0.60
	Model 2	−0.051 (−0.074, −0.028)	<0.001	ref.	−0.02 (−0.042, 0.003)	0.09	0.032 (0.008, 0.055)	0.009	0.41
	Model 3	−0.005 (−0.042, 0.033)	0.81	ref.	−0.038 (−0.076, 0.001)	0.06	−0.033 (−0.073, 0.007)	0.11	0.15

^a^ Median intake (min and max) in mg/day for each tertile. ^b^ (β; 95% CI). We used generalized linear mixed models with the following levels: recruitment center and household. Model 1 is adjusted for sex, age (continuous), and intervention group. Model 2 is as model 1 plus education, smoking status (never, former and smokers), and physical activity in leisure time (sedentary, moderately active, active). Model 3 is as model 2 plus BMI (<30, 30–35, <35), energy intake, intake of carbohydrates, saturated fatty acids, and proteins, alcohol, and glucose-lowering treatment.

**Table 4:** Changes in glucose (mg/dL) and glycosylated hemoglobin (HbA1c) (%) according to tertiles of change in polyphenol intake after one year and stratified by diabetes status at baseline. Results from linear mixed models.

		Non Diabetic Participants (N = 1231, 21%)		Prediabetic Participants (N = 2872, 48%)		Diabetic Participants (N = 1818, 30%)	
		T3 vs. T1	P	T3 vs. T1	P	T3 vs. T1	P
Total polyphenols (mg/d)	Glucose	−0.21 (−1.95, 1.52) ^a^	0.81	−1.16 (−2.25, −0.06)	0.04	−0.83 (−4.95, 3.30)	0.69
	HbA1c	0.011 (−0.040, 0.062)	0.68	0.011 (−0.017, 0.04)	0.44	−0.011 (−0.118, 0.097)	0.85
Total flavonoids (mg/d)	Glucose	−0.99 (−2.72, 0.75)	0.27	−1.66 (−2.75, −0.56)	0.001	−2.24 (−6.39, 1.90)	0.29
	HbA1c	0.025 (−0.028, 0.077)	0.36	−0.009 (−0.037, 0.02)	0.55	−0.014 (−0.122, 0.095)	0.81
Anthocyanidins (mg/d)	Glucose	0.44 (−1.31, 2.19)	0.62	−0.4 (−1.5, 0.7)	0.48	−0.80 (−4.95, 3.35)	0.71
	HbA1c	−0.004 (−0.057, 0.049)	0.87	0.003 (−0.026, 0.031)	0.85	−0.025 (−0.134, 0.083)	0.65
Catechins (mg/d)	Glucose	−0.19 (−1.91, 1.53)	0.83	−0.67 (−1.76, 0.42)	0.23	−2.08 (−6.18, 2.02)	0.32
	HbA1c	0.007 (−0.044, 0.059)	0.79	−0.003 (−0.031, 0.025)	0.84	−0.101 (−0.208, 0.006)	0.06
Proanthocyanidins (mg/d)	Glucose	−0.58 (−2.31, 1.15)	0.51	−1.2 (−2.3, −0.1)	0.03	−2.46 (−6.59, 1.68)	0.24
	HbA1c	0.033 (−0.019, 0.085)	0.21	−0.008 (−0.036, 0.021)	0.58	−0.033 (−0.141, 0.076)	0.56
Flavanones (mg/d)	Glucose	−0.08 (−1.81, 1.66)	0.93	−1.02 (−2.11, 0.08)	0.07	−1.22 (−5.34, 2.89)	0.56
	HbA1c	0.016 (−0.035, 0.068)	0.54	0.007 (−0.022, 0.035)	0.64	0.048 (−0.060, 0.155)	0.39
Flavones (mg/d)	Glucose	−1.03 (−2.78, 0.72)	0.25	−1.27 (−2.39, −0.15)	0.03	−3.02 (−7.24, 1.20)	0.16
	HbA1c	−0.000 (−0.052, 0.051)	0.99	−0.03 (−0.059, 0.000)	0.05	−0.084 (−0.194, 0.025)	0.13
Flavonols (mg/d)	Glucose	−1.39 (−3.13, 0.34)	0.12	−0.95 (−2.05, 0.15)	0.09	−2.46 (−6.58, 1.66)	0.24
	HbA1c	0.009 (−0.042, 0.061)	0.72	−0.036 (−0.065, −0.007)	0.01	−0.148 (−0.256, −0.040)	0.01
Total phenolics acids (mg/d)	Glucose	0.26 (−1.46, 1.97)	0.77	0.48 (−0.62, 1.57)	0.39	0.01 (−4.09, 4.11)	1.00
	HbA1c	0.018 (−0.032, 0.069)	0.48	0.022 (−0.007, 0.05)	0.13	0.006 (−0.101, 0.113)	0.91
Hydroxycinnamic acids (mg/d)	Glucose	0.53 (−1.19, 2.25)	0.54	0.2 (−0.89, 1.3)	0.72	0.14 (−3.97, 4.24)	0.95
	HbA1c	0.02 (−0.031, 0.071)	0.44	0.03 (0.001, 0.058)	0.04	0.003 (−0.104, 0.110)	0.95
Hydroxybenzoic acids (mg/d)	Glucose	−0.13 (−1.90, 1.65)	0.89	0.34 (−0.77, 1.46)	0.55	1.58 (−2.62, 5.78)	0.46
	HbA1c	0.009 (−0.042, 0.061)	0.72	−0.007 (−0.036, 0.022)	0.63	0.034 (−0.077, 0.144)	0.55
Lignans (mg/d)	Glucose	0.1 (−1.64, 1.85)	0.91	−0.62 (−1.73, 0.49)	0.27	−4.83 (−8.99, −0.66)	0.02
	HbA1c	−0.029 (−0.08, 0.023)	0.28	−0.039 (−0.067, −0.01)	0.01	−0.169 (−0.277, −0.062)	0.002
Stilbenes (mg/d)	Glucose	0.61 (−1.26, 2.48)	0.52	−0.21 (−1.39, 0.96)	0.73	−5.51 (−9.87, −1.14)	0.01
	HbA1c	0.022 (−0.034, 0.077)	0.45	−0.021 (−0.051, 0.01)	0.18	−0.079 (−0.192, 0.034)	0.17

^a^ (β; 95% CI). We used generalized linear mixed models with the following levels: recruitment center and household. Regression models are adjusted for sex, age (continuous), and intervention group, education, smoking status (never, former and smokers), physical activity at leisure time (sedentary, moderately active, active), BMI (<30, 30–35, <35), energy intake, and intake of carbohydrates and saturated fatty acids.

## Data Availability

Data described in the manuscript, code book, and analytic code will be made available upon request pending application and approval of the PREDIMED-Plus Steering Committee. There are restrictions on the availability of data for the PREDIMED-Plus trial, due to the signed consent agreements around data sharing, which only allow access to external researchers for studies following the project purposes. Requestors wishing to access the PREDIMED-Plus trial data used in this study can make a request to the PREDIMED-Plus trial Steering Committee chair: jordi.salas@urv.cat. The request will then be passed to members of the PREDIMED-Plus Steering Committee for deliberation.
